# Beyond sensitivity: what are the enabling opportunities of OPM-MEG?

**DOI:** 10.3389/fmedt.2025.1515548

**Published:** 2025-01-21

**Authors:** Timothy P. L. Roberts, Charlotte Birnbaum, Luke Bloy, William Gaetz

**Affiliations:** ^1^ Department of Radiology, Program in Advanced Imaging Research and Lurie Family Foundations MEG Imaging Center, Children’s Hospital of Philadelphia, Philadelphia, PA, United States; ^2^ Department of Radiology, Perelman School of Medicine, University of Pennsylvania, Philadelphia, PA, United States

**Keywords:** optically-pumped magnetometer (OPM), wearable, autism, auditory, pediatric

## Abstract

While optically-pumped magnetometer (OPM) technology offers a number of compelling advantages over its SQUID predecessor for magnetoencephalography (MEG), many studies and viewpoints focus on issues of (i) scalp placement, with commensurate increases in sensitivity to weak magnetic fields and (ii) room temperature operation (without the need for baths of liquid helium to maintain superconducting properties of SQUIDs). This article addresses another unique and tantalizing opportunity—the ability for the OPM array to be “wearable”, and thus to move with the participant. This is critical in adoption of naturalistic paradigms that move beyond “laboratory neuroscience” toward “real world neuroscience”. It is also critically important in application to pediatric populations who cannot or will not remain still during conventional MEG scan procedures. Application to the developing infant brain will be considered as well as application to pediatric neuropsychiatric and developmental disorders, such as autism spectrum disorder. Rather than present solutions, this article will highlight the challenges faced by conventional SQUID-based cryo-MEG and explore the potential avenues for OPM-MEG to make a positive impact to the field of pediatric neuroscience.

## 1 Introduction

While magnetoencephalography (MEG) has developed as a powerful, non-invasive, electrophysiological imaging tool over the past several decades (1), the recent advent of commercially-available optically-pumped magnetometers (OPMs) offers the promise of transformation of the neuromagnetic field (2–4).

Traditional SQUID-based MEG (often termed cryo-MEG because of the need for cryogenic supercooling) has offered considerable clinical utility in the preoperative workup of patients with epilepsy contemplated as candidates for surgery, as well as for guidance of surgical approach trajectory in patients with brain tumors and arteriovenous malformations (AVMs). Given the clear role of MEG in these clinical applications, along with associated North American clinical payment reimbursement, it is perhaps surprising that the number of medical centers that house MEG systems is still rather few. In part, this stems from the relative lack of emergence of practical new clinical indications for the MEG technology, despite its inherent capabilities in terms of functional mapping of brain activity with high spatial, temporal and spectral resolution. The technical limitations of current, typical MEG systems begin to account for this discrepancy: most traditional MEG system utilize superconducting quantum interference device (SQUID)-based detector systems [requiring superconducting temperatures (∼4 K) and associated liquid helium “baths”]. Associated with this is a physical inflexibility in the array of sensors which are “fixed” in the super-cooled helmet and, in order to maintain thermal stability (ensured with surrounding vacuum), the traditional MEG helmet is non-adjustable and, by necessity, “one size fits all” and typically sized to fit the 95th–97th percentile of adult heads.

A major downside of this fixed helmet geometry is that *smaller* heads (for example, infants and young children) will necessarily be distant from the detector sensors encased in the MEG helmet. Since magnetic fields “fall off” or decay according to the square of the distance between source and detector (inverse square law), this leads to an unnecessary and critically-impactful loss of sensitivity to brain activity [e.g., a conventional MEG system might be 4–9x less sensitive to a pediatric brain signal than an adult one, simply by virtue of detector array geometry (5)]. This has led to the development of several dedicated infant/young child MEG systems, such as babySQUID (6), babyMEG (7) and Artemis-123 (8), but, despite their recognized success, these solutions are also limited in their flexibility and may be prohibitively costly. Furthermore, these systems too retain the “problem” of a fixed helmet.

The limitations of the conventional cryo-MEG fixed helmet, however, extend beyond mere loss of sensitivity. This manuscript discusses the possibilities that are enabled by escaping the constraints of the conventional cryo-MEG device, while retaining the spatial, temporal and spectral benefits of magnetic field-based electrophysiology.

### 1.1 Constraints of the cryo-MEG rigid helmet: beyond sensitivity

Beyond compromised sensitivity, recording brain activity using conventional cryo-MEG has additional limitations and sub-optimal characteristics (despite the inherent value of MEG):

1. Intolerance of involuntary motion: a source of artifact, signal loss and source estimation error
2. Intolerance of necessary movement: needed in “realistic” paradigms
3. Anxiety: both the physical appearance of cryo-MEG and the patient instruction to remain motionless can negatively impact the participant's mental state

While impactful, conventional cryo-MEG to-date has been significantly limited in the scope of experimental paradigms, and range of available participant demographics (age, IQ, etc.) to those which are likely to be successfully conducted with only limited (<∼1 cm) head motion. MEG measurement of motor responses (for example) has been almost exclusively limited to ballistic finger movements, or binary (“yes”, “no”) “button press” responses, largely to limit associated, unintended head movement. Thus, the vast majority of what we have come to know about motor function using MEG has been observed through the narrow lens of contrived, artificial “button press” experiments. This practical limitation has significantly restricted the nature and scope of questions we could ask using MEG, placing questions about the neurophysiological basis for motor planning, cognitive control and executive functions of the frontal lobes almost entirely beyond reach. Furthermore, the tantalizing bridge between brain and behavior is often inadequately approached with simple MEG-compatible stimulus delivery and task performance (again, e.g., button press) being correlated with behavioral/clinical assessment performed on a separate occasion, in a separate location under neuropsychologist supervision. The comparative distance between the very specific MEG metrics and the observational metrics of complex behavior performance severely limits the effectiveness of such correlations.

Thus, despite the many attractive opportunities afforded by MEG, populations and studies that suffer from the constraints of conventional MEG include:

1. Infants (who don't understand),
2. Toddlers (who don't cooperate),
3. Children who need to be in a “natural” (non-anxious) state,
4. Studies of “naturalistic” behavior

These, and above, issues are summarized in Table 1, along with a mitigation path through wearable OPM-MEG.

**Table 1:** Challenges to cryo-MEG application and avenues for mitigation through wearable OPM-MEG.

Issue	Paradigm Limits	Patients Affected	OPM solution path
Sensitivity (distance)	Affects all paradigms (inverse square law)	Small ones (infants and young children)	Scalp placement
Involuntary Motion Artifact	Misregistration leads to signal canceling	Toddlers (and all children, especially those with cognitive impairments)	No misregistration btw. head and detector
	Misregistration leads to source error		
	Movement of “on-board” (e.g., dental) metal introduces new noise	Individuals with metallic dental work	Potential “noise” source moves relative to the detector, thereby reducing the associated magnetic field “change” detected
Coupled head and body/extremity movement in order to achieve task	Violation of stationarity artifact	Patients with cerebral palsy, dystonia (9) and other movement-influence conditions	Since detector array is coupled to the head, it can tolerate unavoidable head movements
	Limits to execution of tasks		
Necessary movement not permitted by fixed helmet	Need to keep body in one place	Naturalistic activities: driving, coffee-drinking, BOT-2, Purdue pegboard, “bobbing and weaving”	Since detector array is coupled to the head, it can tolerate needed head and body movements
Anxiety	Influences brain state (elevated & erratic heartrate, movement)	Children who are sensitive to the intimidating environment of cryo-MEG (e.g., anxiety disorders)	OPM-MEG can be made “not scary”—rather, wearable like a bike helmet or a baseball cap
	Some participants even withdraw from cryo-MEG scan session (a recent study of 3yo's with genetic risk of intellectual disability suggests a scanner-intolerance of 15%–20%)		

**Figure 1:**
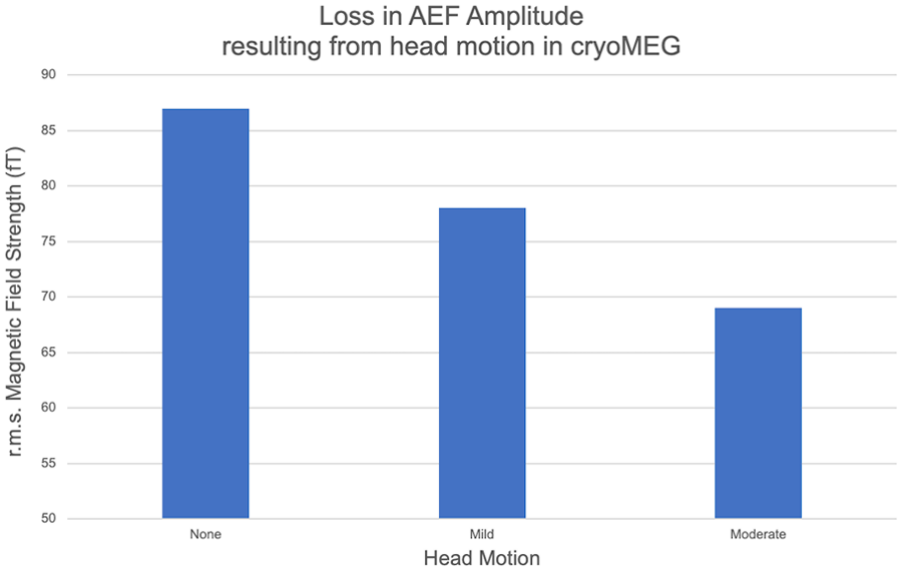
The amplitude of a representative subject's M100 component of the auditory evoked field (AEF) determined under three motion conditions [no motion, mild: single motion 1.3 cm, moderate: continuous motion (max 4.3 cm)] in a conventional cryo-MEG system. The net amplitude of the AEF is seen to decrease as coherent signal averaging is impaired by increasing motion.

Cryo-MEG approaches proposed to enable MEG recording in less-compliant children rely on the ability to track head motion in real time and potentially compensate for mild to moderate (∼1–2 cm) motion. Active fiducial coils attached to the nasion and pre-auricular points carry electrical current at non-harmonically-related frequencies above those considered for brain activity. Coil activity is continually sampled with the coil location determined from the detected coil magnetic field. However, compensation for such trial-to-trial misalignment is not trivial and the consequences of head motion on evoked response amplitude are shown in Figure 1, where signal is progressively lost in evoked responses averaged over multiple trials, in the presence of increasing head motion. This constitutes a milestone to evaluate the potential benefit of OPM-MEG.

## 2 Towards naturalistic opportunities and limitations of cryo-MEG

A few notable attempts have investigated the possibility of exploring more sophisticated/nuanced movements, or recognized the need for movement tasks beyond simple button presses. In-roads to these investigations have been made, probing more complex cognitive interactions but ultimately these endeavors are fundamentally compromised by the requirement for head motion to be minimized in the cryo-MEG rigid helmet, and of course the requirement that not only the head remains still but also the body position remains constant. This is either seldom achievable (in the case of cerebral palsy patients and upper extremity motion in general) or in itself limits the “naturalism” of the paradigm. Nonetheless, we acknowledge the potential of these approaches, even with conventional cryo-MEG acquisition hardware, and imagine how yet more efficacious use could be made of these paradigms if the head-stationarity constraints were to be relaxed in a wearable MEG solution.

### 2.1 Simulated driving

Simulated driving provides not only a window into the cognitive neuroscience of driving itself, but also a paradigm for naturalistic investigation of visuo-motor integration. By defining characteristic “events” in driving such as “accelerating”, “braking” etc., analysis of MEG signals allows inference of event-related oscillatory dynamics, arising from both the motor cortex as the node for mechanical output as well as centers of executive function and cognitive control.

However, a limitation of studying simulated driving with conventional rigid MEG hardware is the unrealistic requirement for constraining head motion, eliminating driving relevant behavior such as shoulder/mirror checking, and constraints on orienting to unexpected events in the driving scene (e.g., children playing at the roadside). As a result, only “head forward” stimuli can be presented, significantly reducing the “reality” of the simulation. We envision an immediate opportunity for wearable MEG allowing *realistic* behavior in this *realistic* paradigm.

### 2.2 Target-touch motor task (TTMT)

The study of motor function in clinical populations can yield fascinating information about the scope of neural plasticity in the context of developmental challenges. Unfortunately, *clinical* motor impairments can, in themselves, present significant *technical* obstacles: for example, patients with focal dystonia (9) and cerebral palsy (CP) may be unable to comply with conventional motor task constraints, expectations and button-press hardware. Alternative strategies that can accommodate the movement of affected digits/limbs are needed. Motivated by these challenges, Gaetz et al. have developed a dedicated movement task for MEG recording accommodating full extremity motion in a visually-cued event-related paradigm (10), involving the presentation of interactive reach-to-target stimuli, soliciting movements of the upper arm in contradistinction to a simple conventional button press paradigm. In the Target-Touch Motor Task (TTMT), whole-arm movements to visual targets are captured using MEG compatible infra-red hardware. In addition, targets can be hit using any hand posture, from an individual finger, a clenched fist or atypical posture. As a result, TTMT game play can accommodate a wide variety of postures allowing the study of the neural correlates of motor impairment with MEG.

However, recording using cryo-MEG even with this novel paradigm is limited. Postural stability during reaching is challenging in children with CP, leading to increased head motion in direct relation with arm reaching (11, 12). As a result, during MEG recordings, we have observed substantially greater overall head motion in children with CP.

## 3 OPMs as a basis for wearable MEG

**Figure 2:**
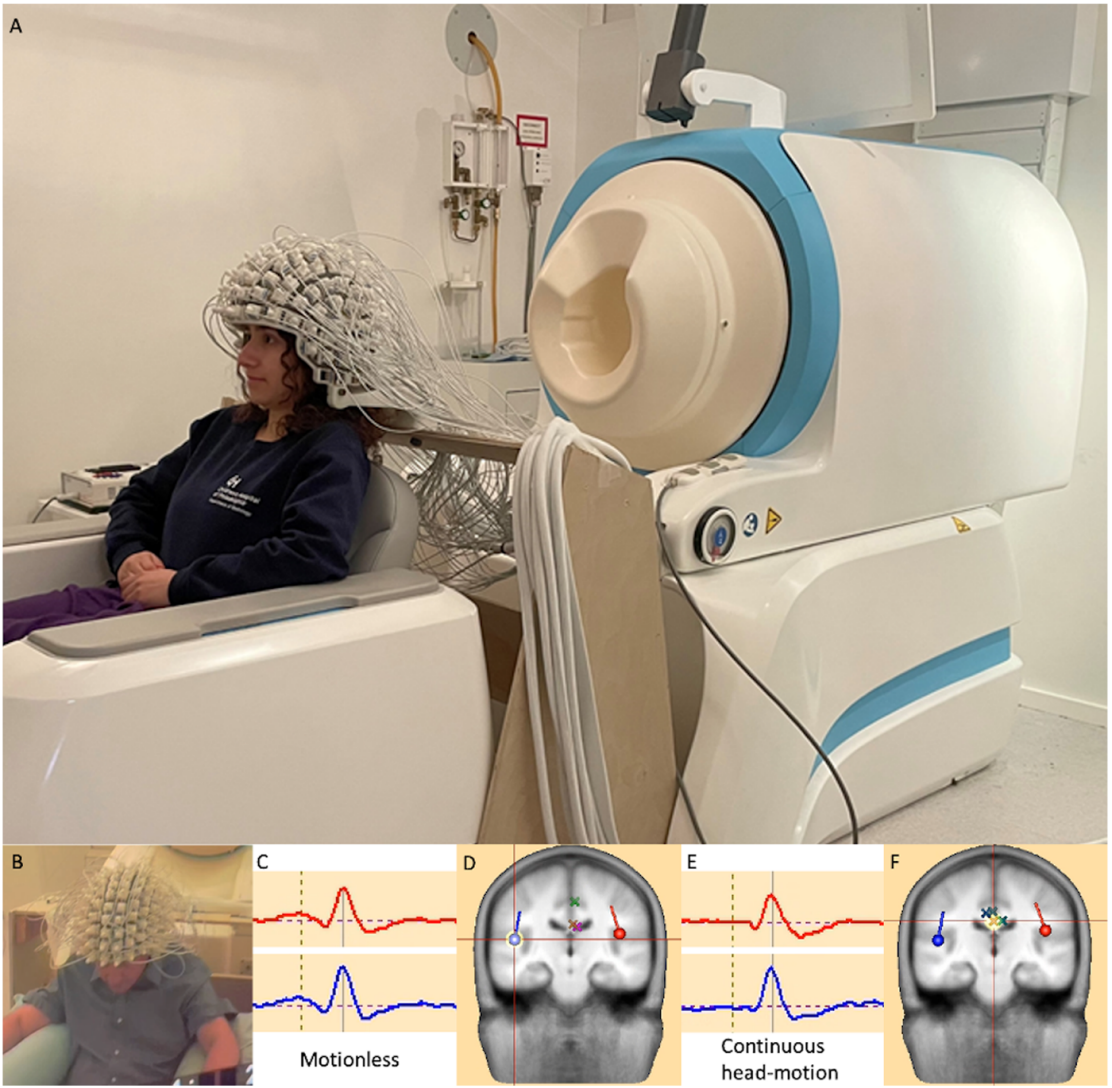
**(A)**: A head-mounted OPM-MEG array offers visibly-appreciable difference to the conventional rigid helmet of a cryo-MEG. It potentially will offer the benefits of magnetic electrophysiology with a form factor more closely approximately an EEG array, with increased sensitivity (through scalp placement of detectors) and motion tolerance (through the coupling of the detector array and the participant's head), mitigating motion “artifacts” as well as tolerating realistic movements. [Written release was obtained for the photographic image]. The lower panel presents feasibility data for a single healthy adult subject **(B)**, during an AEF paradigm using an OPM-MEG array in **(C,D)**: a motionless condition and **(E,F)**: with data obtained during continuous 5–10 cm head movement. Bilateral source modeled auditory responses and localizations [e.g., crosshair on right hemisphere dipole source in **(D)**] look highly comparable between conditions, with environmental noise contributions “projected away” as pseudo- (or nuisance) sources, identified by simple principal component analysis (PCA), implemented in BESA Research 7, (BESA Gmbh, Graefelfing, Germany) and modeled as dipole sources [marked as X's—see crosshairs in **(F)**].

The recent emergence of OPMs as an alternative magnetic field sensor to the SQUID employed in conventional cryo-MEG devices offers the promise of addressing some of the above challenges and realizing some of the above opportunities (2–4, 13, 14). By obviating the need for superconducting technology, it is possible to escape the confines of the rigid helmet required for supercooled (liquid helium/vacuum placement) sensors and thus release three specific constraints—(i) proximity: with no need for thermal shielding and fixed placement within a rigid helmet, OPM sensors can be placed much closer to the surface of the head, with corresponding gain in magnetic field sensitivity (15); (ii) weight/rigidity/movement, removing the constraint for a dewar filled with liquid helium, the constraints of fixed-position helmet are removed, paving the way for head-based “wearable” arrays of sensors, that can move with the subject's head (analogous to EEG or fNIRS) and exhibit head movement tolerance that will mitigate artifact *and* enable difficult pediatric implementation as well as tapping yet unrealized potential from naturalistic paradigms, all while (iii) diminishing participant anxiety and emotional discomfort from the device appearance and the instruction to remain (typically unnaturally) motionless. As such, OPM-MEG may be considered to fill a space in the sensor continuum between conventional cryo-MEG and high density (HD)-EEG, potentially offering the benefits of magnetic field recording (e.g., superior source modeling afforded by the physics of magnetism vs. electricity in the setting of the tissue media of the brain, skull and scalp) with the convenience and form-factor of a wearable device (more akin to the HD-EEG net commonly employed). Although, OPM-MEG may nevertheless still exhibit motion-related artifacts, hardware and software approaches to ameliorate these are under ongoing development (introduced below, although a comprehensive treatment is beyond the scope of the present Perspective article), and present feasibility data (see Figure 2) offer promise that the potential of wearable MEG with OPMs may indeed be realizable.

In essence the possibilities enabled by OPM-based “wearable” MEG can be considered as:

1. “Routine” paradigms in “non-routine” participants/patients
2. “Non-routine” paradigms

### 3.1 Substantial equivalence: OPM arrays vs. cryo-MEG

In order to support the potential opportunities of OPM-based MEG recording, it is of paramount importance to demonstrate that substantially equivalent recordings can be made between OPM and conventional cryo-MEG systems. Moreover, it is important to demonstrate that the promise of substantially equivalent data quality can be realized using an OPM array *even during moderate free head movement* compared to that derived from a more typical motionless recording.

### 3.2 Noise rejection in OPM-MEG

The adoption of OPM technology necessarily requires a noise cancelling methodology, fundamentally due to the difference between magnetometers and gradiometers (and not *cryo*-MEG vs. *OPM*-MEG, *per se*) in which the magnetometer remains sensitive to environmental noise sources (e.g., but not limited to, 60 Hz power line) which the gradiometer substantially attenuates due to its far-field, common-mode, rejection. In OPM recordings, these noise contributions can overwhelm the underlying neuronal activity and may not be amenable to simple spectral filtering. Several promising methods have been proposed. For example, Seymore et al., (2022) detail several possible interference suppression techniques that may be valuable for addressing the relative abundance of environmental noise commonly observed during OPM measurements (16). While the nuances of noise-rejection algorithm methods are beyond the scope of this manuscript, and optimal choice thereof will likely depend on details of experimental paradigms and dependent variables, it is reassuring that simple application of e.g., principal component analysis, PCA, or linearly constrained minimum variance (LCMV) beamforming may allow recovery of underlying neural signal that is (a) substantially equivalent to the cryo-MEG gradiometer recording and (b) still demonstrates feasibility even in the setting of considerable mead motion.

## 4 Future clinical applications

Beyond the inherent advantage of improved sensitivity of OPM-MEG, it is the *tolerance of head motion*, that will confer two critical benefits opening up new areas of application: (1) reduction/elimination of motion “artifact”, that is the enabling of current experiments in populations currently underserved—infants, toddlers and children with behavioral and cognitive impairments and (2) the emerging conduct of naturalistic behavioral paradigms or even during clinical behavioral assessment.

The latter, in particular, will enable the tighter coupling of brain and behavior, no longer comparing estranged button-press brain activity with sophisticated clinical observation of motor behavior, but actual brain recording during the measurement of clinically relevant behavior. As an example consider the Purdue Pegboard task (17) or BOT-2 assessment (18), the neural correlates of which are probably not best proxied with a simple repetitive button press, as has been attempted hitherto. The specific method to “capture” events of interest during a BOT-2 task, for example, will depend on the presence of clear, repeatable events of interest (analogous to how e.g., braking events are identified in simulated driving 19). The scope of event definition is likely to range from “in-context” button presses (e.g., touch-sensitive transducers embedded in the pegboard) towards eventually moving to digital motion analysis from synchronized high frame rate video capture.

### 4.1 Epilepsy, general anesthesia and long-term monitoring

Tolerance of movement is not infinite, but limited by the linearity of the OPM detector. Environmental noise may present extant gradients and other inhomogeneities within the MSR, ultimately limiting tolerable displacement. Motion (of the sensors) itself may also compromise sensitivity. Thus, a narrow volume (of dimension ∼10s of cm), may define the operating regime of the wearable MEG in most environments (see e.g., Figure 2). Ongoing development of large-scale magnetic field gradient “room” coils to partially compensate for magnetic field heterogeneity promises to further expand this “sweet spot” of OPM linearity (20). Advances in sensor design, specifically tri-axial configurations and closed-loop “field-adaptive” sensors are similarly under development, increasing tolerance to field inhomogeneity and motion, as well as cross-axis projection errors (CAPE) collectively (21, 22).

So what applications can be enabled by a few 10's of cm of movement tolerance? Here we offer two tantalizing opportunities expected to be afforded by wearable MEG, and might demonstrate immediate clinical impact, as well as a third (virtual reality, VR) which might provide a research framework for new endeavors.

#### 4.1.1 Obviating the need for general anesthesia (GA) in presurgical pediatric epilepsy evaluations

While the information provided (to date) by cryo-MEG in the presurgical evaluation of patients with epilepsy is well-documented (23, 24), many younger patients (and even older patients with some intellectual/behavioral disabilities) are unable to comply with the requirement to hold still and thus would be precluded from cryo-MEG recording. However, the information delivered by MEG is considered so clinically valuable that it is common practice for such patients to undergo cryo-MEG under general anesthesia (GA) to eliminate head motion confounds. Indeed, over several thousand clinical MEG scans over the past two decades at our institution, we have conducted 20%–25% under GA. Nonetheless, the limitations of conducting MEG scans under GA are several: (i) the anesthesia itself acts to dampen brain activity, at least theoretically compromising the sensitivity of the recordings, (ii) anesthesia is associated with increased logistical challenges, costs and, potentially, morbidities, and (iii) conducting scans under anesthesia precludes any active task performance and therefore limits concomitant functional mapping to somatosensory cortex activation using electrical median nerve stimulation; no motor or language mapping is feasible. One promise of OPM-MEG, is the opportunity to conduct clinical MEG recordings in such epilepsy patients. If OPM-MEG proves feasible in such patients, with equivalent clinical yield to cryo-MEG with GA, then the requirement for GA will be obviated, also enabling a full assessment of somatosensory, motor and language areas. Initial clinical research suggest that clinically valuable MEG recording can indeed be made with OPM-arrays (25); expanded comparison studies vs. cryo-MEG with GA are a warranted next step.

#### 4.1.2 Long-term monitoring (LTM) of preoperative patients with epilepsy

Related to the use of MEG without GA, lies another opportunity for OPM-MEG to shift the paradigm of pre-operative workup of patients with epilepsy. Conventional practice currently involves extended periods (up to ∼1week) of scalp (phase 1) or intracranial (phase 2) EEG electrode placement with continuous synchronized video recording, to capture “ictal” seizure events and their electroencephalographic correlates. Of key importance is that the seizure events captured during phase 1 or 2 video telemetry are associated with head/body movement, but this movement may well be measured in tens of cm and may thus occur within a “sweet spot” of magnetic field homogeneity that would permit OPM recordings to continue to be feasible. As such, one could contemplate a novel “long term monitoring unit” in which an OPM-MEG array is “worn” (and even “slept in”) for an extended period (several days) and whereby seizure events are captured on synchronized video and MEG, allowing anatomic localization by source modeling of the magnetic field without requiring surgical implantation.

### 4.2 Virtual reality (VR) stimulus paradigms

VR environments have become a mainstay of modern video gaming entertainment, transforming the commercial video market, allowing participants to experience synthetic real-world environments and interactions, all within an “operating” enclosure of the order of 1 m radius. Beyond gaming, major social media channels are exploring methods with which to expand the VR experience to a broader domain of social interactions, perhaps transforming the “social” experience in a fashion analogous to the transformation of telephonic communication enabled by video-calls. Early studies (26) have offered preliminary data suggesting the compatibility of OPM-MEG recording of participants experiencing VR stimulation/interaction. Again, the range of *actual* physical motion incurred during *virtual* reality immersion is rather limited (∼1 m) and thus potentially can exist within a sweet-spot of OPM-linearity.

## 5 Summary

The advent of OPM-MEG systems promises to free the clinical researcher/neuroscientist from the shackles of conventional cognitive neuroscience, namely overly simplistic paradigms, as well as head motion intolerance, currently limiting the participant populations that can be served. OPMs can thus be viewed as opening up MEG inclusivity for infants, babies and children unable to comply with the physical constraints of cryo-MEG, as well as offering the prospect of scanning “children at play” as well as more tightly coupling synchronized quantitation of brain and behavior. Thus, beyond sensitivity, it is the motion tolerance of OPM-MEG that may likely enable future research and clinical endeavor.

## Data Availability

The original contributions presented in the study are included in the article/Supplementary Material, further inquiries can be directed to the corresponding authors.
